# Development of a Promising Fish Model (*Oryzias melastigma*) for Assessing Multiple Responses to Stresses in the Marine Environment

**DOI:** 10.1155/2014/563131

**Published:** 2014-03-03

**Authors:** Sijun Dong, Mei Kang, Xinlong Wu, Ting Ye

**Affiliations:** Key Lab of Urban Environment and Health, Institute of Urban Environment, Chinese Academy of Sciences, Number 1799 Jimei Street, Xiamen 361021, China

## Abstract

With the increasing number of contaminants in the marine environment, various experimental organisms have been “taken into labs” by investigators to find the most suitable environmentally relevant models for toxicity testing. The marine medaka, *Oryzias melastigma*, has a number of advantages that make it a prime candidate for these tests. Recently, many studies have been conducted on marine medaka, especially in terms of their physiological, biochemical, and molecular responses after exposure to contaminants and other environmental stressors. This review provides a literature survey highlighting the steady increase of ecotoxicological research on marine medaka, summarizes the advantages of using *O. melastigma* as a tool for toxicological research, and promotes the utilization of this organism in future studies.

## 1. Introduction

Estuaries and coastal waters are contaminated by high levels of anthropogenic pollutants [1], creating an urgent need for ecotoxicological studies of marine pollution. The ecotoxicological characteristics of pollutants in saltwater and freshwater environments are different. The parameters of seawater are significantly different from those of freshwater (i.e., salinity, density, buoyancy, pH, ionic strength, and dissolved oxygen (DO)), and these differences impact the ecotoxicological characteristics of the pollutants, such as the packing fraction and size, the distribution of the contaminants in liquid and solid phases, and the bioaccumulation of the contaminants [2–4].

In addition, the studies of the organisms living in the two different environments have also presented different results. Although *Oryzias latipes* (freshwater fish) and *Oryzias melastigma* (seawater fish) are closely related, their branchial FXYD domain-containing ion transport regulator (FXYD) proteins exhibit divergent expression patterns [5]. *Kif7* is not expressed in *O. melastigma* but is highly expressed in the brain of zebrafish, which is a freshwater fish [6]. There is an inverse correlation between the muscle water contents (MWC) and salinity in *O. latipes*; however, the two parameters are not related in *O. melastigma* [7]. Exposure to perfluorooctane sulfonates (PFOS) shortened the hatching time and increased the hatching rate of *O. melastigma* but had the opposite effects in zebrafish [8–10]. These differences illustrate that ecotoxicological results from freshwater environments cannot be directly applied to the marine environment. At present, aquatic toxicological research is largely carried out under freshwater environmental conditions, and research in the marine environment is urgently needed.

The biologic impact of toxic pollutants on fish is an important area of study in ecotoxicology. Fish models, such as zebrafish (*Danio rerio*), tilapia (*Oreochromis niloticus*), and rainbow trout (*Oncorhynchus mykiss*), have been widely used for ecotoxicological studies in the freshwater environment. Although some estuarine species, for example, *Corophium acherusicum*, *Enteromorpha linza*, and *Ctenogobius giurinus*, can be used for the study of ecotoxicology in marine environments, the research still lags well behind that in freshwater environments, and problems such as species specificity and the lack of genetic information in these species do exist.

*O. melastigma*, also named* O. dancena* or Indian medaka, has many advantages as a fish model in marine toxicological research. This review summarizes the advantages and research findings of marine toxicological studies using *O. melastigma* and encourages further investigation of ecotoxicology in the marine environment using this fish model.

## 2. Advantages of **O. melastigma ** as a Research Model in Toxicological Studies

*O. melastigma* originates from the coastal waters and fresh waters of Pakistan, India, Myanmar, and Thailand. In classification, *O. melastigma* and *O. latipes* belong to the order Beloniformes, family Adrianichthyidae and genera* Oryzias*. The embryo of this species has been identified as an important tool for toxicology investigations by the regime of ILSI Health and Environmental Sciences Institute (HESI). As a fish model, it shares many advantages as follows.

1. *O. melastigma* is small in size (4.5 to 23 mm) and has a short generation time (2-3 months). These characteristics make it available to culture on a large scale under laboratory conditions (30‰ artificial seawater, 28 ± 1°C, and in a 14 h light: 10 h dark cycle). The relatively large eggs and transparent color simplify experimental observations and operations, such as observing developmental changes during each stage of growth [11].
2. *O. melastigma* has distinct sexual dimorphism, and the morphology of the anal fin is very prominent approximately 1 month after hatching, rendering it highly desirable for gender studies [12]. Researchers have recommended that future risk evaluation of immune-modulatory chemicals must include parallel assessment of both genders. This makes *O. melastigma*, owing to its characteristics of distinct gender dimorphism and the presence of sex-determining *Dmy* gene of its homologous species *O. latipes*, suitable for toxicity evaluation [13].
3. *O. melastigma* possesses strong environmental tolerance. This organism is capable of adapting to a wide range of temperatures; thus, mutants can be derived that are conveniently temperature sensitive [14]. *O. melastigma* has the ability to survive in aquatic environments with a wide range of salinity. Although *O. latipes* can adapt to varying salinity environments to some degree, the adaptive capacity of *O. latipes* is lower than that of *O. melastigma*, which can thrive in water of varying salinity ranging from 0 to 35 ppt [1].
4. The eggs and larvae of *O. melastigma* are sensitive to many environmental pollutants. If the specific sensitive gene responding to pollutants or other environmental stresses can be identified at the molecular level, then environmental pollution can be quickly identified. The molecular staging of* O. melastigma* embryos, focusing on the heart, pectoral fin, brain, eye, pancreas, muscle, liver, and neuron system, has been fully described [15].
5. Studies of *O. latipes* in anatomy, physiology, and other aspects have been increasingly extensive and systematic, and the genome sequences of *O. latipes* have been completed. Many common characteristics exist between *O. latipes* and *O. melastigma* in phylogeny; thus, the brackish *O. melastigma* can serve as a good marine fish model for developmental studies by utilizing the resources developed from *O. latipes*. The corresponding genetic chip information of *O. melastigma* has been acquired which makes it convenient for the study of *O. melastigma* [1, 14, 16]. Additionally, homologous species could be fully used for comparative biology, in a similar manner to *Drosophila*, for which the genome analysis of multiple species has greatly promoted the study of comparative biology [14, 17].

All of these advantages enhance the potential of *O. melastigma *to be a competent model organism in marine ecotoxicology.

## 3. The Research Background of *O. melastigma* in Molecular Biology 

Sharing a high degree of similarity, most of the research findings of the congeneric species of *O. melastigma*, such as *O. latipes*, could be applied to *O. melastigma* mostly. Notably, even though *O. melastigma* is similar to the other medaka species, some differences still exist. For example, *omChgh* is characterized by eight exons and seven introns, while the second isoform of the *Chgh* gene has only seven exons in the *O. latipes* genome [6, 18, 19]. *Dlx2* is expressed only in the telencephalon and diencephalon of *O. melastigma*, while it is also expressed in the rhombencephalon of *O. latipes* [1]. *O. latipes* and *O. melastigma* share completely identical peptide sequences but bear very different glycan structures [19]. This phenomenon suggests that further exploration of the marine medaka genome and proteome is needed [20].

### 3.1. The Research Background of *O. melastigma* Genes

A substantial number of molecular biological studies for *O. melastigma* are being conducted. The complete mitochondrial genome of *O. melastigma* has been obtained from the genome data sequenced by next-generation sequencers [21]. A batch of organ-specific molecular markers have also been identified, such as the makers for brain, eyes, heart, liver, and muscle [15]. These markers can be used to indicate the developmental status of specific organs, and their abnormal expression can be used to indicate the toxicity of pollutants on organ development. Chen et al. [1] analyzed the expression of 11 organ-specific expression genes during each period of embryonic development by *in situ* hybridization (ISH) and determined that 8 of the 11 genes are similar to those expressed during the embryonic development of zebrafish and *O. latipes*.

In addition to the above specified genes of organ development, some functional genes in different tissues have been analyzed as well (Table 1). Some immune-related genes, including complement-related genes and inflammation-related genes, have been analyzed. Bo et al. used suppression subtractive hybridization (SSH) to identify differentially expressed immune genes in the liver of *O. melastigma* infected with *Vibrio parahaemolyticus* [30]. Based on an NCBI BLAST search of the 1279 sequenced clones in the SSH libraries, 396 genes were identified, and 38 were involved in the immune process. Additionally, genes involved in cellular metabolism, biological regulations, general response to stimuli, transport processes, signal transduction, and cellular component organization were obtained [30]. Some genes related to metabolism, osmoregulatory and cardiac development in *O. melastigma* have also been submitted. Whole *omCyp* genes were registered at the GenBank database. To date, various *Cyp* gene families have been identified. The transcript profiling of whole *omCyp* genes has been finished for *O. melastigma* exposed to water accommodated fractions (WAFs) of Iranian crude oil [25, 33].

Second generation high-throughput sequencing technology has greatly enhanced the ability to obtain genetic information. Huang et al. extracted RNA from *O. melastigma* following exposure to pollutants during various developmental periods and used Illumina high-throughput sequencing to obtain 6 GB data. They performed bioinformatics analyses and identified a large number of toxicology-related genes, thus providing a broad molecular basis for further toxicological investigations [27].

Differentially expressed genes can be largely obtained in fish after exposure to pollutants using gene chip technology. Chinese scholars have constructed a dedicated gene chip for *O. melastigma*, which contains 180 genes related to cell division, detoxification reactions, hypoxia response, oxidative stress, apoptosis, growth, sex determination, gonadal differentiation, and reproductive hormone secretion [35]. This chip includes the most common marker genes for toxicological studies and can be used effectively for gene screening with differential expression. Using newly developed sequencing technology (Illumina RNA-Seq) and digital gene expression (DGE) technology, a total of approximately 145 thousand unigenes were obtained with 565 bp of unigene N50 [27], which were further enriched in various molecular pathways involved in the response to PFOS exposure and related to neurobehavioral defects, mitochondrial dysfunction, and the metabolism of proteins and fats.

### 3.2. The Research Foundation of *O. melastigma* Proteins

The detection of protein expression levels requires corresponding antibodies. Because of the conservation of homologous proteins, antibodies have certain commonalities in allied species. The antibody library of zebrafish has been relatively completed; thus we can use them to directly screen for the specific antibody that reacts with the homologous protein in *O. melastigma*, avoiding the tedious processes of antibody preparation. Through immunohistochemical assay (IHCA) screening of whole embryos, 17 types of zebrafish antibodies can cause specific immune reactions with *O. melastigma*. These antibodies have a close relationship with the development of nerve, heart, and brain, providing a basis for toxicological research on protein levels [15, 16]. In addition, mouse anti-human TERT monoclonal antibody mAb476 can specifically combine with the TERT protein of *O. melastigma* [16].

The tissue distribution of the protein expression in *O. melastigma* under various environment stresses has been partly finished intuitively by WB, IHCA, and matrix-assisted laser desorption/ionization tandem time-of-flight mass spectrometry (MALDI-TOF/TOF MS) (Table 2). The expression of the TERT protein in the cytoplasm and nucleus of *O. melastigma* can be quantified by Western blotting (WB) [41]. Kong et al. observed the TERT protein expression of *O. melastigma* in the testis, ovary, muscle, brain, gill, intestine, kidney, and liver of adult fish using IHCA [16]. Proliferating Cell Nuclear Antigen (PCNA) is the protein marker reflecting cell proliferation, which can be detected by means of IHCA in* O. melastigma*. Experimental results showed a significant correlation between PCNA and TERT in transcriptional and translational expression levels [11]. PCNA detection can also reflect the spatial and temporal characteristics of *O. melastigma* embryonic development [15].

Proteomics refers to the research method of identifying protein characteristics on the large-scale level, and it has become one of the hot spots of aquatic toxicology [39]. Quantitative proteomic analysis demonstrated that hepatotoxicity caused by Hg might involve oxidative stress, cytoskeleton impairment, and energy metabolism alteration, highlighting that the fish liver might be an important target for Hg attack. And proteins such as cathepsin D, GST, and peroxiredoxin-1 responding to Hg treatment in a dose-dependent manner could be used as potential biomarkers of aquatic Hg monitoring [38]. Exposure to PbTx-1 resulted in the alteration of the protein expression involved in cell structure, macromolecule metabolism, neurotransmitter release, and the distribution of signal transduction which may help explain the damage mechanisms of aquatic toxins in fish [36].

## 4. Utilization of *O. melastigma* in Toxicological Studies

*O. melastigma* has been used as a research model for assessing multiple responses to stresses of organic chemicals, inorganic chemicals, detrimental organisms, and environmental stress (Table 3). The toxicity responses of *O. melastigma* are different from some species under environmental stresses, which may even have a totally opposite effect (Table 4).

### 4.1. Toxicological Studies for Organic Chemicals

The choriogenin of teleost fish is considered to be part of the structural interlayer of chorionic precursor cells, which are sensitive to estrogenic contaminants. It increased the expression of the egg-shell precursor protein gene in the liver when exposed to a high concentration of 17*β*-Glycol and 17*α*-ethinyl estradiol [34]. The *Chgh* and *Chgl* of *O. melastigma* are sensitive to exposure to estradiol and nonylphenol, and the response of the male fish is more sensitive compared to the female. This indicates that the two genes can be used as sensitive biomarkers to detect pollution levels of estrogen contaminants in the marine environment [34].

The WAF exposure induced CYP-involved detoxification effects but reduced CYP-involved steroidogenic metabolism in the marine medaka. As well-characterized biomarkers of toxicants exposures, *omCyp1a* and *omCyp1b* were highly induced following WAF exposure [25, 43]. Some previous studies have shown potentially synergistic effects after coexposure of *O. melastigma* embryos to CYP1A inhibitors and PAH-type CYP1A inducers [43]. The acute aquatic toxicity of some seawater organisms exposed to polycyclic aromatic hydrocarbons (PAHs) in the laboratory is summarized in Table 5. Distinctly, *O. melastigma* showed high tolerance to PAHs compared to other species. The heart elongation (heart tube) of *O. melastigma* embryos and heart deformities of these juvenile fishes have been recommended as potential biomarkers of the existence of PAH pollution by Mu et al. [43].

Studies quantified the endogenous expression of all six complement system genes including *C1r/s*, *Mbl2*, *CfpF2*, *C3*, and *C9*, in the liver of marine medaka and found that the expression levels were higher in males than in females. BDE-47 exposure downregulates the expression of all six genes in males, while in females the expression of *Mbl2*, *Cfp*, and *F2* mRNAs was upregulated and *C3* and *C9* remained stable with exposure time and dose. These results indicate that the future direction for fish immunotoxicology should include parallel assessment for both genders [29]. Two hepcidins in *O. melastigma* play a complementary role in the innate defense system. Gender specificity should be taken into consideration in immunotoxicological studies in time and extent of induction of the two hepcidin genes in infected *O. melastigma* [48].

PFOS has estrogenic activity and endocrine-disruptive properties that elicit transcriptional responses on POPs-related pathways in a stage-specific manner [61–63]. The marine biological toxicity of PFOS was systematically studied by Dong et al. using *O. melastigma* [10, 23, 26–28]. Their results showed that exposure to PFOS could induce the hatching enzyme both at transcriptional and enzymatic activity levels and further lead to decreases of average hatching time and increases of the average hatchability of *O. melastigma* embryos, which in turn induced the mortality of the larvae hatched from exposed embryos. All of these effects were dose dependent [10]. They also found that PFOS is toxic to the development of the cardiovascular system of *O. melastigma*, affecting the expression of cardiac development-related genes, morphological development, and function of the heart in the marine medaka [23].

Some research has also been conducted in their laboratory with embryos exposed to low concentrations of bisphenol A (BPA). The result showed that the expression of heart development-related genes and inflammation-related genes in *O. melastigma* was altered, the body length and width decreased, and the larvae exhibited inflammation foci in the heart ventricles [31].

### 4.2. Toxicological Studies for Inorganic Chemicals

Subacute toxicity experiments with ambient concentrations of pollutants are often closer to environmental value and thus have great significance in toxicological evaluation. In evaluating the toxicity of ZnO, researchers evaluated the subacute toxicity of two zinc oxides on the expression of SOD, MT, and HSP70 in *O. melastigma* and found that the two zinc oxides show differences in the induction of three proteins [40]. In the toxicity assessing, double-wall carbon nanotubes (DWNTs) (10 mg/L) following ultrasonic treatment inhibited the growth of *O. melastigma* larvae [46].

*O. melastigma* is also used in the evaluation of heavy metal toxicity. The 96 h LC50s of this fish following exposure to Cu^2+^, Cd^2+^, Hg^2+^, Cr^6+^, Pb^2+^, and Zn^2+^ are shown in Table 6, from which we could determine that *O. melastigma* has a strong sensitivity to metal stress compared to other marine species. Toxicity detection of *O. melastigma* for copper, tributyltin (TBT), and five commonly used antifouling fungicides, including s-triazine, diuron, pyrithione zinc, copper pyrithione, and chlorothalonil, indicate that the 96 h LC50 of this fish's tolerance for copper, s-triazine, and diuron is at the level of mg/L, while others are *μ*g/L [91]. Exposing fertilized eggs and newly hatched *O. melastigma* juveniles to Cd^2+^, Hg^2+^, Cr^6+^, and Pb^2+^ significantly reduced the hatching ability of the embryos and the heart rates above a certain concentration [47]. Metal accumulation of inorganic mercury in the liver and brain of *O. melastigma* induced oxidative stress, cytoskeletal reorganization and/or disruption, dysfunction in metabolism, protein modification, signal transduction, and other related functions [37, 38].

### 4.3. Toxicological Researches for Detrimental Organisms

The median lethal time (LT50) of *O. melastigma* is treated as an indicator of pollutant toxicity for toxicological comparison and correlation analysis. In addition, the acute toxicity test can provide the appropriate dose for the study of molecular toxicological mechanisms, such as the determination of the 24 h LC50 value in *O. melastigma* exposed to brevetoxins (PbTxs). These concentrations of PbTxs can be determined in follow-up proteomics studies [36].

ISH showed that *Vibrio parahaemolyticus* would induce the expression of hepcidin genes in the nuclei and cytoplasm of liver cells of *O. melastigma* [48]. It is also used to characterize the toxicity of the toxins generated by *Chattonella marina* and *Karenia brevis*. Test fishes exposed to the toxins generated by *C. marina* exhibit hyperventilation, while those exposed to the toxins generated by *K. brevis* exhibit hypoventilation [49]. With the assistance of the proteomic approach combined with other methods, the toxicological mechanism of aquatic toxins in marine organisms will be elucidated easily and conveniently [36].

### 4.4. Toxicological Studies for Environmental Stress

*O. melastigma* can serve as a marine fish model for assessing molecular responses to stresses in the marine environment. Hypoxia upregulates *omTert* expression via omHIF-1 in the liver and testis of nonneoplastic fish [22]. Significant changes were observed in the transcription, translation, cell proliferation, and apoptosis level of TERT in the liver of *O. melastigma* exposed to hypoxic conditions for 3 months [16]. Anoxic conditions can increase the expression of *Tert* in the liver and testicular tissue of *O. melastigma*, which is mediated by anoxia-induced factor-1 [24]. The expression of leptin receptor gene exhibits tissue specificity when exposed to hypoxia, and this gene was identified as a sensitive marker gene for a hypoxic environment [22].

The experimental animal, marine medaka, is suitable for studying the mechanism of hypoosmoregulatory. Studies show that branchial om*Nkcc1a* mRNA levels are induced significantly with an increase in environmental salinities. Salinity-dependent expression of *Nkcc1a* is in the branchial mitochondria-rich (MR) cells of *O. melastigma*, which suggests a critical role in hypoosmoregulatory endurance of this fish [32]. Studies have also indicated that *O. latipes* exhibited better hypoosmoregulatory ability, while *O. melastigma* exhibited better hyperosmoregulatory ability. These results support the hypothesis that the lowest branchial NKA activities of these two species were found in the environments that have similar salinities to their natural habitats [7].

## 5. Conclusion

*O. melastigma* have biological characteristics such as small size, high fecundity, short life cycle, sexual reproduction, and distinctive life stages that would allow their use as a marine fish model. Additionally, their ease of cultivation facilitates the use of *O. melastigma* in independent laboratories. The availability of knowledge on their sensitivity towards inorganic and organic compounds and the increasingly complete knowledge on their genes and proteins will also enhance the potential of *O. melastigma* as suitable models in marine aquatic ecotoxicology and toxicogenomics. Researchers have demonstrated the potential application of *O. melastigma* as an ideal marine test fish for marine pollution assessments and ecotoxicological studies of organic chemicals, inorganic chemicals, microorganism, and environmental stress in relation to cardiac toxicity, hepatotoxicity, neurotoxicity, ecotoxicity, immunotoxicity, and so forth.

*O. melastigma* can also serve as a model marine fish for assessing multiple *in vivo* molecular responses to stresses in the marine environment. *O. melastigma* showed high tolerance to PAHs and strong sensitivity to metal stress compared to other species. The heart elongation of *O. melastigma* embryo and *omChgh* and *omLepr* expression are used as potential biomarkers to indicate PAH mixtures contamination or an oil spill, estrogenic chemicals in the marine environment, and growth and/or endocrine disruption in this marine fish, respectively. The expression of the leptin receptor gene, which was identified as a sensitive marker gene for hypoxia environment, exhibits tissue specificity in *O. melastigma*. We may be able to develop biomarkers for more specific adverse effects that can be used for both ecotoxicology and human health risk assessment because of the high degree of evolutionary conservation among vertebrates [92].

Although some toxicological research has been conducted using this small fish species as a model, there is still much to be studied. Fortunately, transcriptome analyses and proteomic approaches, along with new methodologies in *O. melastigma*, such as gene knockdown, gene overexpression, gene chip technology, second-generation high-throughput sequencing technology, RNA-Seq, and DGE technology, can be expected to further accelerate the knowledge of the toxicological mechanisms of aquatic toxins in marine animals in the future. Demonstrating and understanding toxicity mechanisms in *O. melastigma* that are common between humans and fish and wildlife are necessary if we are to integrate findings from laboratory and ecotoxicology studies with human health risk assessment.

## Figures and Tables

**Table 1:** Expression of the cloned genes of* O. melastigma* in different tissues under various environmental stresses.

Functions	Genes	Exposed tissues	Exposed to	References
Reference genes	*18S, Rpl7,* and **β*-actin *			

Hypoxia-responsive	*Telomerase reverse transcriptase (Tert) *	Ovary, liver, testis, kidney, gill, brain, spleen, intestine, eye, muscle, and skin	Hypoxia	[16, 22]
	*Hypoxia-inducible * *factor-1*α** * (Hif 1*α*) *	Liver, testis	Hypoxia	[22]
	*Erythropoietin (Epo) *	Liver, testis, and embryos	Hypoxia, PFOS	[22, 23]
	*Leptin receptor (Lepr) *	Liver, gill, heart, kidney, gill, brain, spleen, intestine, eye, muscle, ovary, and testis	Hypoxia	[24]
	*Hemoxygenase-1 (Ho) *	Liver, gill, and heart	WAFs, Hypoxia	[24, 25]

Immune toxicity	*Glutathione peroxidase (Gpx) *	Embryos	PFOS	[26]
	*Catalase (Cat) *	Embryos	PFOS	[26]
	*Uncoupling protein 2 (Ucp2) *	Embryos	PFOS	[26, 27]
	*Cyclooxygenase-2 (Cox2) *	Embryos	PFOS	[26]
	*Peroxisome proliferator-activated receptors(Ppars): Ppar*α*, Ppar*β*, * and *Ppar*γ**	Embryos, whole fish	PFOS, WAFs	[25, 26, 28]

Complement-related genes	*Lectin, mannose-binding 2 (Mbl2) *	Liver	PBDE-47	[29]
	*Cyan fluorescent protein (Cfp) *	Liver	PBDE-47	[29]
	*Complement component: C1r/s, C3, C9, C3-2, C4, C1q, C5, C8, C1 inhibitor *	Liver	PBDE-47, *Vibrio parahaemolyticus *	[29, 30]
	*Prothrombin (F2) *	Liver		[30]
	*Complement factor: Hf, Bf *	Liver	*Vibrio parahaemolyticus *	[30]
	*Hepcidin (Hep): Hep1, Hep2 *	Liver, spleen, gill, intestine, ovary, testis, brain, and embryos	*Vibrio parahaemolyticus *	[30]
	*Mannose-binding lectin-associated serine protease (Masp) *	Liver	*Vibrio parahaemolyticus *	[30]

Inflammation-related genes	*Tumor necrosis factor-*α* (Tnf*α*) *	Embryos	PFOS, BPA	[26, 31]
	*Interleukin (Il): Il1*β*, Il8 *	Embryos	PFOS, BPA	[26, 31]
	*CC chemokine eotaxin-1 (Ccl11) *	Embryos	BPA	[31]
	*Superoxide dismutase (Sod) *	Embryos	PFOS, BPA	[26, 31]

Osmoregulatory mechanism	Na^+^/K^+^ *-ATPase (Nka) *	Gill, embryos	BPA, SW (35‰), BW (15‰), and FW (0)	[7, 31, 32]
	Na^+^, K^+^, 2Cl^−^ * cotransporter (Nkcc): Nkcc1a, Nkcc1b, * and *Nkcc2 *	Gill, liver, testis, intestine, ovary, brain, muscle, kidney, heart, Fin, and eye	SW (35‰), BW (15‰), and FW (0)	[32]
	*FXYD domain-containing ion transport regulator (Fxyd): Fxyd5, Fxyd6, Fxyd7, Fxyd8, Fxyd9, Fxyd11,* and *Fxyd12 *	Gill, intestine, kidney, brain, eye, liver, and caudal fin	SW (35‰), BW (15‰), FW (0)	[5]

Cardiac development-related genes	*NK2 transcription factor related 5 (Nkx2.5) *	Embryos	PFOS, BPA	[23, 31]
	*Cyclooxygenase (Cox): Cox1, Cox2 *	Embryos	PFOS, BPA	[23, 31]
	*ATP synthase *	Embryos	PFOS	[23, 27]
	*Bone morphogenetic protein (Bmp4) *	Embryos	PFOS, BPA	[23, 31]
	*Fibroblast growth factor 8 (Fgf8) *	Embryos	PFOS, BPA	[23, 31]
	*GATA-binding protein 4 (Gata4) *	Embryos	PFOS, BPA	[23, 31]
	*Leptin receptor (Lerp) *	Embryos	BPA	[31]
	*SET and MYND domain containing 1 (Smyd1) *	Embryos	PFOS	[23]

Metabolisms	*Cytochrome P450 (Cyps) *	Liver, gill, embryos, intestine, and ovary	PFOS, WAFs	[25, 28, 33]
	*Aldehyde dehydrogenase (Aldh) *		WAFs	[25]
	*Glutathione S-transferase (Gst): Gsta, Gstk, Gsto, Gstt, * and *Gstz *		WAFs	[25]
	*Sulfotransferase (Sult): Sult1, Sult2 cytosolic, Sult2b1, Sult2b2, Sult3 cytosolic, Sult3-like, Sult4a1, * and *Sult6b1 *		WAFs	[25]
	*UDP-glucuronyltransferases (Ugts): Ugt1b, Ugt2a, Ugt2a2, Ugt2a3, Ugt2b33, Ugt2b3-like, Ugt5a1, * and *Ugt5g1 *		WAFs	[25]
	*Hydroxysteroid dehydrogenase (Hsd): 3*β*-Hsd, 11 *β*-Hsd, * and *17*β*-Hsd *		WAFs	[25]
	*Aryl Hydrocarbon Receptor (Ahr): Ahr1, Ahr2 *	Embryos, whole fish	PFOS, WAFs	[25, 28]
	*5 *α*-reductase (Srd5a) *		WAFs	[25]
	*Steroidogenic acute regulatory protein (Star) *		WAFs	[25]
	*ATP-binding cassette (Abc): Abcb1, Abcc2, Abcc3, Abcc4, * and *Abcg2 *		WAFs	[25]
	*Heat shock protein (Hsp): Hsp10, Hsp22, Hsp27, Hsp30, Hsp60, Hsp70, Hsp75, Hsp90a, Hsp90*β*, Hsp*β*7, * and *Hsp*β*11 *		WAFs	[25]
	*Choriogenin H and L (Chgh and Chgl) *	Liver, embryos, and larvae	PFOS, E2, EE2, BPA, and NP	[28, 34]
	* Kinesin superfamily7 (Kif7) *	Brain, kidney, liver, muscle, ovary, and testicle		[6]
	*Aryl hydrocarbon receptor nuclear translocator (Arnt) *	Embryos	PFOS	[28]
	*Vitellogenin (Vtg) *	Embryos, liver, gill, intestine	PFOS	[28]
	*Estrogen receptor (Er) *	Embryos	PFOS	[28]
	*Horiolysin H and L* (*Hce and Lce) *	Embryos	PFOS	[10]

Notes: 2,2′,4,4′-tetrabromodiphenyl ether (PBDE-47), bisphenol A (BPA), polycyclic aromatic hydrocarbons (PAHs), sea water (SW), fresh water (FW), brackish water (BW), 17*β*-Estradiol (E2), 17*α*-ethinylestradiol (EE2), 4-nonylphenol (NP).

**Table 2:** Expression of proteins in different tissues of *O. melastigma* under various environmental stresses.

Related functions	Proteins	Expression tissues and exposure condition	References
Cell structure	Histone-binding protein RBBP4	Gill (Br)	[36]
	Gelsolin	Gill, brain (Br)	[36]
	Krt4 protein	Gill (Br)	[36]

Oxidative stress response	Hemoglobin beta chain	Gill (Br)	[36]
	Histone H3	Gill (Br)	[36]
	Glial fibrillary acidic protein	Brain (Br)	[36]
	Keratin 15 [KRT15]	Brain (Br), liver (Hg)	[36, 37]
	Zgc: 65851	Brain (Br)	[36]
	Type I cytokeratin, enveloping layer [CYT1]	Brain (Br), liver (Hg)	[36, 37]
	Myosin light chain 2	Brain (Br)	[36]
	Tropomyosin alpha-3 chain	Brain (Br)	[36]
	*α*-Tubulin 1	Liver (Hg)	[37]
	Keratin 8	Liver (Hg)	[37]
	*α*-Actin	Liver (Hg)	[37]
	Keratin 18	Liver, brain (Hg)	[37]
	*β*-Actin	Liver, brain (Hg)	[37]
	Type I keratin-like protein	Liver (Hg)	[37]
	Lamin type B	Liver (Hg)	[37]
	Krt5 protein	Brain (Hg)	[37]
	Type II basic cytokeratin	Brain (Hg)	[37]
	Keratin K10 [KRT10]	Liver (Hg)	[38]
	Novel protein similar to vertebrate plectin 1 [PLEC]	Liver (Hg)	[38]
	Peroxiredoxin 4	Liver (Hg)	[38]
	Peroxiredoxin 6	Liver (Hg)	[38]
	Glutathione S-transferase [GSTR]	Liver (Hg)	[38]
	SOD [Cu-Zn]	Liver (Hg)	[38]
	Aldehyde dehydrogenase 1 family, member A2	Brain (Hg)	[38]
	Aldehyde dehydrogenase, mitochondrial	Brain (Hg)	[38]
	Peroxiredoxin-2 [PRDX2]	Liver (Hg)	[38]
	Natural killer enhancing factor	Liver (Hg)	[37]
	Peroxiredoxin-1 [PRDX1]	Liver (Hg)	[38]
	DJ-1 protein [DJ-1]	Liver (Hg)	[38]
	Cathepsin D [CTSD]	Liver (Hg)	[38]
	proliferating cell nuclear antigen [PCNA]	Testis, muscle, kidney, liver Cheek, brain, intestine, and ovary embryo during each development period (H)	[16, 39]
	Telomerase Reverse Transcriptase [TERT]	Testis, brain, muscle, gill, intestine, kidney (N), and liver (H)	[16]
	superoxide dismutase [SOD]	Whole fish (Z)	[40]
	Metallothionein [MT]	Whole fish (Z)	[40]
	heat shock protein 70 [HSP70]	Whole fish (Z)	[40]

Metabolism	ApoA-IV4	Gill (Br)	[36]
	Aldose reductase	Gill, brain (Br)	[36]
	Pyruvate carboxylase	Brain (Br)	[36]
	Dpysl5a protein	Brain (Br)	[36]
	Triosephosphate isomerase	Brain (Br)	[36]
	Enolase	Brain (Br)	[36]
	Glutamine synthetase	Brain (Br, Hg)	[36, 37]
	Isovaleryl coenzyme A dehydrogenase	Brain (Br)	[36]
	Glyceraldehyde 3-phosphate dehydrogenase	Brain (Br)	[36]
	Homogentisate 1,2-dioxygenase	Liver (Hg)	[37]
	Alanyl-tRNA synthetase, cytoplasmic	Liver (Hg)	[37]
	Dihydrolipoamide S-acetyltransferase	Liver (Hg)	[37]
	Adenosylhomocysteinase	Liver (Hg)	[37]
	Pyruvate dehydrogenase E1 component subunit alpha, somatic form, mitochondrial	Liver (Hg)	[37]
	Brain-type fatty acid binding protein	Liver (Hg)	[37]
	Methionine adenosyltransferase-like	Liver (Hg)	[37]
	S-formylglutathione hydrolase	Liver (Hg)	[37]
	Apolipoprotein A1	Brain (Hg)	[37]
	Pyruvate kinase	Brain (Hg)	[37]
	Dihydropyrimidinase-related protein 5	Brain (Hg)	[37]
	Dihydropyrimidinase-like 2	Brain (Hg)	[37]
	Enolase 1, (alpha)	Brain (Hg)	[37]
	Creatine kinase, brain b	Brain (Hg)	[37]
	Total glutathione [GSH]	Whole fish (W)	[25]
	Glutathione *S*-transferase [GST]	Whole fish (W)	[25]
	Sulfotransferase [SULT]	Whole fish (W)	[25]
	Superoxide dismutase [SOD]	Whole fish (W)	[25]
	Glutathione reductase [GR]	Whole fish (W)	[25]
	Glutathione peroxidase [GPx]	Whole fish (W)	[25]
	Catalase, CAT	Whole fish (W)	[25]
	ATP synthase subunit d, mitochondrial [ATP5H]	Liver (Hg)	[38]
	Electron-transferring-flavoprotein dehydrogenase [ETFDH]	Liver (Hg)	[38]
	Electron transferring flavoprotein subunit alpha, mitochondrial [ETFA]	Liver (Hg)	[38]
	Pyruvate dehydrogenase (lipoamide) beta [PDHB]	Liver (Hg)	[38]
	Phytanoyl-CoA dioxygenase domain-containing protein 1 [PHYD1]	Liver (Hg)	[38]
	Delta3,5-delta2,4-dienoyl-CoA isomerase, mitochondrial [ECH1]	Liver (Hg)	[38]
	Phosphorylase [PYGB]	Liver (Hg)	[38]
	Formimidoyltransferase-cyclodeaminase [FTCD]	Liver (Hg)	[38]

Signal transduction	Putative transient receptor protein 2	Gill (Br)	[36]
	Myosin regulatory light chain 2	Gill (Br)	[36]
	FXYD domain-containing ion transport regulator	Gill (S)	[5]
	NKCC1a-like protein	Gill (S)	[32]
	NKA *α*-subunit	Gill (S)	[7, 32]
	Grancalcin	Gill (Br)	[36]

Protein modification	Myosin light chain 2	Gill (Br)	[36]
	Calreticulin, like 2	Gill (Br)	[36]
	Transforming protein RhoA	Brain (Br, Hg)	[36, 37]
	Calmodulin	Brain (Br)	[36]
	Annexin 4	Liver (Hg)	[37]
	14-3-3E1 protein	Liver (Hg)	[37]
	14-3-3 protein	Liver (Hg)	[37]
	Annexin A13	Brain (Hg)	[37]
	Cytosolic nonspecific dipeptidase	Liver (Hg)	[37]
	Proteasome alpha 1 subunit	Liver (Hg)	[37]
	HSP-90	Brain (Hg)	[37]

Other function related	Chaperonin containing TCP1, subunit 8 (theta)	Brain (Hg)	[37]
	Beta-synuclein	Brain (Br)	[36]
	SH3-domain GRB2-like endophilin B2	Brain (Br)	[36]
	Complement component C3-1	Liver (Hg)	[37]
	Carbonic anhydrase 1	Brain (Hg)	[37]
	ATPase, H+ transporting, V0 subunit D isoform 1	Brain (Hg)	[37]
	Transferrin	Brain (Hg)	[37]
	Eukaryotic translation initiation factor 3, subunit 2 beta [EIF3S2]	Liver (Hg)	[38]
	Histone H4	Liver (Hg)	[38]
	Ependymin [EPD]	Liver (Hg)	[38]
	GammaN1 crystallin [CRYGN1]	Liver (Hg)	[38]

Notes: the abbreviations in parentheses mean the protein expression in the environment of exposure to normal (N), hypoxia (H), brevetoxins (Br), HgCl_2_ (Hg), salinity (S), nZnO (Z), and WAFs of Iranian crude oil (W).

**Table 3:** Utilization of *O.  melastigma* as a research model for toxicological studies.

Responsive to	Toxicological research about	Age of fish	Exposure concentration and time	Main works	Main conclusions	References
Organic chemicals						
WAFs	CYP1A-involved detoxification mechanism	3-week-old fish and adults	2.5, 5, 10, 20, 40, 60, 80, and 100% WAF for 24 h; 5% for 6, 12, 24, 48, 72, and 96 h	Transcript profiling of whole *omCyp* genes, enzyme activity and steroid hormones assay, *omCyp1a* mRNA expression in different tissues during different developmental stages, and effects of *β*-NF, BaP, and WAF on expression of *omCyp1a *	WAF induced CYP-involved detoxification mechanism but reduced steroidogenic metabolism; *omCyp1a* would be associated with the initiation of the cellular defense systems.	[25, 33]
PBDE-47	Immune-modulatory effects	Three-month-old	290 and 580 ng/day from 2 dpf to hatching	Correlation between BDE-47 body burden and complement gene expression (RT-PCR) in different genders	Genes studied were gender dependent (males > females); BDE-47 is not biotransformed in marine medaka.	[29]
	Maternal transfer	2- and 3-month-old	1.3 ± 0.2 *μ*g/day for 21 days	Accumulation of PBDE 47 in 2-month-old fish and maternal transfer of PBDE 47 from adult female medaka to eggs	PBDE 47 transfer is associated with lipid mobilization during egg production.	[42]
PFOS	Mitochondrial dysfunction	Embryos	0.25 and 1 mg/L from 2 dpf to 6 dpf	Sequence the RNA mixtures using Solexa/Illumina RNA-Seq at various developmental stages and after various types of exposure, and DGE and qRT-PCR analysis for relative gene expression	The mitochondrial dysfunction appears to be involved in multiple toxicological effects of PFOS on *O. melastigma* embryos.	[27]
	Precocious hatching	Embryos	1, 4, and 16 mg/L from 2 dpf to hatching	Record the time for hatching, hatching rate and mortality of fry hatched within a week, and hatching enzymatic activity and RT-PCR analysis for gene expression	PFOS induced the hatching enzyme, leading to the precocious hatching of embryos and the decrease of larvae survival.	[10]
	Endocrine-disruptive effect	Embryos	1, 4, and 16 mg/L for 2 dpf, 4 dpf, and 10 dpf, respectively	The mortality and malformation rates, the transcriptional responses of the ER, AHR, and PPAR pathways to PFOS by RT-PCR, and quantification of PFOS in exposure solutions and medaka embryos	PFOS has estrogenic activity and endocrine-disruptive properties and could elicit gene responses in a stage-specific manner.	[28]
	Cardiac toxicity	Embryos	1, 4, and 16 mg/L for from 2 dpf to hatching	Cardiac morphology, heart rates and the SV-BA distance of the heart was measured; RT-PCR analysis of gene expression profiles was conducted.	PFOS affected the development and function of the heart in the marine medaka embryos.	[23]
	Immunotoxicity	Embryos	0, 1, 4, and 16 mg/L from 2 dpf to hatching	PFOS body burden, survival rates, and growth parameters of fish larvae during 17 dph, liver histological examination, and gene expression in fish larvae after LPS exposure for 12 h at 27 dph	The immunosuppression effects caused by PFOS could lead to functional dysfunction or weakness of the immune system in the fish larvae.	[26]
BPA	Cardiac toxicity	Embryos	200 *μ*g/L for 2 dpf-incubation	Heart beat rate, SV-BA distance of embryos, body length and width, histology, and BPA-induced inflammation-related genes and heart-related genes	BPA induced cardiac toxicity of the *O. melastigma* embryos.	[31]
PAHs (ANF, Pyr, Phe, and BaP)	Developmental malformations	Embryos	Different PAHs for 18 days	Deformity assessment, heart rate, heart elongation, hatch rate, and EROD and Caspase-3/7 activity assays of embryos exposed to PAHs with or without 100 *μ*g/L ANF	Inhibition of CYP1A, EROD, and Caspase-3/7 activities can be used as indicator in the ecological early warning and PAHs detection.	[43, 44]
Estrogen (E2, EE2, NP, and BPA)	Estrogenic pollutants	Sexually mature	E2, EE2 (1, 10, 100, and 500 ng/L); NP, BPA (1, 10, 100, and 200 *μ*g/L) for 7 days	E2-inducible choriogenins expression in embryos and yolk-sac larvae by end-point PCR; effects of EE2, BPA, and NP, respectively, on *omChgh* and *omChgl* expression by RT-PCR	The rapid inducibility (within 24 h) of *omChgh* by E2 during early developmental stages was found to be more estrogen sensitive than *omChgl. *	[34]
Benzotriazole	Reproductive effect	3-month-old	0.01, 0.1, and 1 mg/L for 4 and 35 days	Benzotriazole can induce *Vtg* and *Cyp19a* gene expression but inhibits the *Cy1a1* gene expression (qPCR analysis).	Benzotriazole had adverse potential on the endocrine system.	[45]

Inorganic chemicals						
DWNTs	Ecotoxicity data of DWNTs	48 h posthatching	10, 50, and 100 mg/L for 14 days	Mortality and total length of medaka fish larvae over 14 days exposed to different concentrations of stirred and sonicated double-walled carbon nanotubes.	So-DWNTs are more toxic than st-DWNTs; the dispersion method and size of aggregations should be considered in DWNT toxicity testing.	[46]
nZnO	Sublethal toxicities	<24 h	4 and 40 mg/L ZnO for 96 h	Stress responses in fish after acute exposure (SDS-PAGE)	nZnO did not display the same toxicity as ZnO towards the fish.	[40]
HgCl_2_	Hepatotoxicity and neurotoxicity	Weighing 0.5 ± 0.05 g	1000 *μ*g/L for 8 h; 1 or 10 *μ*g/L for 60 d	Protein expression profile in liver and brain exposed to HgCl2 (MALDI-TOF/TOF MS) and mercury accumulation and damaged liver ultrastructure in medaka	Hg hepatotoxicity might involve oxidative stress, cytoskeleton impairment, and a dysfunction in metabolism.	[37, 38]
Cd^2+^, Hg^2+^, Cr^6+^, and Pb^2+^	Toxic effects of heavy metals	Embryos and larvae	96 h and 14 d	The mortality, heart beat rate, and malformation rates	The fish species has relatively high sensitivity to heavy metal stress.	[47]

Detrimental organisms						
*Vibrio parahaemolyticus *	Immunotoxicity	5-month old	6 × 105 cfu/fish for 6 h, 24 h and 48 h	qPCR analysis of the complement genes in liver; age-, tissue-, and gender-differences in the expression of *hepcidin*; *hepcidin* expression in hepatocyte by ISH	*O. melastigma* can serve as a model to understand the basic biological processes related to immune function.	[30, 48]
*K. brevis*: PbTx-1	Neurotoxicity	Adult	0, 6, 8, 10, 12, 16 and 18 *μ*g/L for 24 h; 6 *μ*g/L for 2 days	Algal toxicity (toxic symptoms, 24 hour mortality, 1/LT50) and its supernatant, MeOH and TCM extracts of *O. melastigma*; changes in protein profiles in medaka gill and brain exposed to PbTx-1	*K. brevis*-induced hypoventilation response in medaka; the down-regulation of several proteins involved in cell protection.	[36, 49]
*C. marina *	Ichthyotoxins of *C. marina *	4–8 months-old	10,000 cells/mL for 0, 24, 48 and 60 h	Algal cell density, growth rate, their toxicity (toxic symptoms, 24-hour mortality, 1/LT50) and its supernatant, MeOH and TCM extracts to *O. melastigma *	Fish susceptibility to *C. marina* is related to its growth rate, but not to cell density; *C. marina* developed the hyperventilation response of the fish.	[49, 50]

Environmental stress						
Hypoxia	Hypoxia-responsive	4-week old adult	1.8 ± 0.2 mg O_2_/L for 3 months; 12 weeks 1.8 mg O_2_/L for 24, 48 and 96 h	Adult male fish were processed for ISH and IHC; volume density indices of omTERT mRNA and protein, PCNA and TUNEL signals in liver hepatocytes after chronic exposure to hypoxia; expression of *Tert*, *Hif 1*α**, *Epo*, *Lepr*, and *Ho* in tissues by RT-PCR	Hypoxia upregulates omTERT expression via omHIFhif-1 in liver and testis and the omLepR omLEPR expression demonstrated its independent control in endocrine and peripheral tissues.	[16, 22, 24]
	Ichthyotoxins of *C. marina *	4–8 months old	7 mg/L, 6.0 mg/L and 1 mg/L DO for 60 h	Oxygen consumption rate, threshold lethal DO and correlation between body weight and survival time of marine medaka inside the sealed syringe	Fish susceptibility to *C. marina* is related to the susceptibility of the fish to hypoxia.	[50]
Salinity	Osmoregulatory mechanism	2.50 ± 0.30 cm	SW (35‰), BW (15‰), FW (0) for Three weeks or 1 month	Plasma osmolality, MWC, Na^+^/Cl^−^ concentration, time course, NKCC1a-like protein expression, NKA activity, NKA-IR cell activity, NKA *α*-subunit mRNA and protein expression in gills in response to hypoosmotic challenge; salinity effects on multiple *Fxyd* mRNA and FXYD11 protein abundance; co-immunoprecipitation of NKA with FXYD11 and the localization of *Fxyd11* mRNA in gill sections in freshwater-acclimated marine medaka	The expression pattern of branchial *Fxyd11* was similar to that of *Nka*α** in the *O. latipes*, but non-correlated expression patterns were observed in the *O. melastigma* at both the mRNA and protein levels; the lowest NKA activities were found in the environments with salinities similar to their natural habitats.	[5, 7, 32]
	Ichthyotoxins of *C. marina *	4–8 months old	70‰ seawater and natural seawater.	The LT50 of marine medaka at different ages (4–8 months-old) exposed to 70‰ hypersalinity (70‰-SW)	Fish susceptibility to *C. marina* is not related to its tolerance to hypersalinity stress.	[50]

Notes: days postfertilization (dpf); days posthatching (dph); sinus venosus-bulbus arteriosus (SV-BA); lipopolysaccharides (LPS); *β*-naphthoflavone (*β*-NF); benzo[a]pyrene (BaP); phenanthrene (Phe); pyrene (Pyr); methanol (MeOH); chloroform (TCM); quantitative polymerase chain reaction (qPCR).

**Table 4:** Comparative toxicity of *O.  melastigma* and other species under various stresses.

Exposing to	*O. melastigma *	Other species	References
PFOS	Hatched in advance and hatching rate increased.	Hatch was delayed and hatching rate was not affected or decreased in zebrafish.	[8–10]
	Ke in the larvae ranged from 0.04/d to 0.07/d.	Ke ranged from 0.053 to 1.700 L/kg/d in blood, kidney, liver and gall bladder and from 0.02 to 0.23/d in carcass and liver concentrations in rainbow trout (*Oncorhynchus mykiss*).	[51–53]
	Did not alter *Epo *	Led to high mortality in zebrafish	[23]

Phe, Pyr, and BaP	NOEC values were 50, 25, and 10 *μ*g/L, respectively.	NOEC values were 10, 50, and 1.8 *μ*g/L, respectively, in the water flea (*Tigriopus japonicus*).	[54]

E2	The mRNA level of *3Bhsd* (steroidogenic enzymes) was increased.	Decreased the production of 11-KT and mRNA levels of steroidogenic enzymes in zebrafish and decreased the production of testosterone in human	[55–57]

DWNTs	Growth inhibition was observed at 10 mg/L for so-DWNTs but not for st-DWNTs.	Population growth was reduced to 0.1 mg/L for so-DWNTs and 10 mg/L for st-DWNTs in the water flea.	[23]

nZnO	Lack of change was observed in the SOD activities.	SOD activities were decreased for the first few days but recovered soon in *O. latipes* and were also significantly depleted in mouse embryo fibroblast cells, more toxic in Skeletonema costatum and Thalassiosira pseudonana, and less toxic in Elasmopus rapax and the water flea.	[40]

Cercariae	Did not infect	Infected in liver and kidneys of *Channa punctatus*, infected in the muscles of *Cliona orientalis*, and did not infect in *Puntius sophore* and *Gambusia affinis *	[58]

Salinity	Prefers hypoosmotic conditions	Prefers hyperosmotic conditions in Javanese medaka (*Oryzias Javanicus*)	[11, 59]
	MWC was constant with the increase of salinity in *O. melastigma*.	MWC was decreased with the increase of salinity in *O. latipes*.	[7]

Hypoxia	HAS was not present.	HAS was identified in zebrafish and *Fugu*.	[60]
	*3Bhsd* and *Cyp19a* mRNA expression upregulation	*3Bhsd* and *Cyp19a* mRNA expression was reduced in zebrafish.	[55]

Notes: the elimination rate constant (Ke); No Observed Effect Concentration (NOEC); 11-ketotestostrone (11-KT); HIF-1 ancillary sequence (HAS).

**Table 5:** Acute toxicity data (96 h LC50/EC50) of seawater organisms exposed to PAHs.

Scientific name	LC50/EC50	PAHs (*μ*g/L)			References
		Phe	Pyr	BaP	
*Corophium acherusicum *	LC50	310	49	—	[54]
*Neomysis awatschensis *	LC50	130	—	—	[54]
*Tigriopus japonicus *	LC50	546	174	3.46	[54]
*Neomysis awatschensis *	LC50	—	15.2	—	[54]
*Nitzschia closterium *	EC50	71.5	56.8	51	[54]
*Enteromorpha linza *	EC50	2070	209	286	[54]
*Oncorhynchus mykiss *	LC50	3200	2000	—	[64]
*Acanthogobius lactipes *	LC50	295	—	—	[54]
*Ctenogobius giurinus *	LC50	—	13.1	—	[54]
*O. melastigma *	LC50	6399	3127	5705	[54]
*Sparus macrocephalus *	LC50	800	—	—	[54]
*Strongylocentrotus intermedius *	LC50	520	—	—	[54]
*Hemicentrotus pulcherrimus *	LC50	—	1.056	1.56	[54]

Notes: median lethal concentration (LC50); median effective concentration (EC50).

**Table 6:** Acute toxicity data (LC50, mg/L) of metals in various species.

Species	Exposure ages	LC50 (mg/L)						References
		Cu^2+^	Cd^2+^	Pb^2+^	Cr^6+^	Hg^2+^	Zn^2+^	
*Argopecten ventricosus *	Juvenile	—	0.396	0.830	3.430	—	—	[65]
*Chironomus furens *	Larvae	52.8	0.3	0.3	0.3	0.03	4.5	[66]
*Chironomus plumosus *	Larvae	42.6	0.4	8.2	1	0.3	9.5	[66]
*Chironomus riparius *	Adult	0.043	0.021	—	—	—	—	[67]
*Cynoglossus semilaevis *	Postlarvae	0.025	0.178	1.026	—	0.045	1.18	[68]
*Duttaphrynus melanostictus *	Larvae	0.03	0.3	4.2	—	—	4.2	[69]
*Echinogammars olivii *	Adult	0.25	—	0.62	—	—	1.30	[70]
*Farfantepenaeus paulensis *	Postlarvae	—	0.83	—	—	—	3.31	[71]
*Fundulus heteroclitus *	Postlarvae	1.7	18.2	188	—	0.068	129.5	[72–74]
*Hyalella azteca *	Adult	0.21	0.013	—	—	—	—	[67]
*Hexagenia* spp.	Adult	0.073	7.82	—	—	—	—	[67]
*Liza vaigiensis *	Postlarvae	—	3.7	138	—	0.0835	—	[72]
*Lutjanus argentimaculatus *	Juvenile	—	—	98	20.1	0.38	—	[75]
*Menidia menidia *	Juvenile	—	6.3	—	91	0.112	—	[75, 76]
*Oreochromis niloticus *	Juvenile	0.80	—	—	—	0.82	—	[77]
*O. latipes *	Postlarvae	—	5.6	—	12.4	—	—	[72]
*O. melastigma *	Postlarvae	7.3	1.12	>20	1.456	0.097	43	[78]
*Pagrosomus major *	Postlarvae	0.31	5.6	—	—	—	3.6	[79, 80]
*Palaemon elegans *	Adult	2.52	—	5.88	—	—	12.3	[70]
*Penaeus indicus *	Postlarvae	0.8204	—	7.22	—	—	—	[81, 82]
*Penaeus monodon *	Postlarvae	—	2.28	5.77–7.28	—	—	3.02	[83, 84]
*Penaeus penicillatus *	Larvae	—	3.025	—	—	—	4.267	[85]
*Poecilia reticula *	Juvenile	2.36	17.71	—	43.4	—	—	[86]
*Penaeus setiferus *	Postlarvae	0.0308	—	—	—	0.017	—	[87, 88]
*Priopidichthys marianus *	Juvenile	—	—	140	31	0. 35	—	[72]
*Rivulus marmoratus *	Postlarvae	1.4	21.1	85.3	14.3	—	147.9	[72, 74]
*Sphaeroma serratum *	Adult	1.98	—	4.61	—	—	6.12	[70]
*Sparus macrocephalus *	Larvae	0.2	0.3	—	—	—	1.8	[80]
*Stenocypris major *	Adult	0.0252	0.0131	0.5262	—	—	1.1898	[89]
*Tubifex tubifex *	Adult	0.16	0.87	—	—	—	—	[67]
*Zebrafish *	Adult	0.174	6.497	116.432	181.09	0.14	44.48	[86, 90]
